# Comparison of Two Quality Analysis Checklists Used to Appraise Studies Regarding the Assessment of Auditory Processing Disorder in Older Adults

**DOI:** 10.1055/s-0044-1792083

**Published:** 2025-05-29

**Authors:** Vipin Ghosh, Asha Yathiraj, Darshan Devananda

**Affiliations:** 1 Department of Audiology, JSS Institute of Speech and Hearing, Mysuru, Karnataka, India; 2 Department of Speech Language Pathology, JSS Institute of Speech and Hearing, Dharwad, Karnataka, India

**Keywords:** SQAC, MDBC, meta-analysis, systematic review

## Abstract

**Introduction** A meta-analysis of published articles is usually done using standard scales and checklists. Several such scales and checklists are reported in the literature. However, there is little information regarding their utility so one can select the most appropriate one, especially in the field of audiology.

**Objective** The current study aimed to compare a quality analysis carried out using the standard quality assessment criteria (SQAC) for evaluating primary research papers from a variety of fields', and the Modified Downs and Black Checklist (MDBC) for a set of articles in the area of auditory processing deficits (APDs) in older adults.

**Methods** Two published checklists suitable for the field of audiology (SQAC and MDBC) were compared for a quality analysis of articles on APD in older adults. The two checklists were compared after categorizing their items into five subsections. Two audiologists rated the articles according to both checklists.

**Results** The interrater reliability was found to be good for both checklists. Significant differences between the checklists were observed for specific subsections. However, there was no significant correlation between the two checklists.

**Conclusion** It is inferred that the selection of an appropriate quality assessment checklist depends on the objective of the study. If the aim of a quality analysis study is to differentiate articles based on their overall caliber, or primarily based on the subsections, SQAC is recommended. However, if the aim is to distinguish research articles primarily based on the control of variables, or differentiate intervention-based studies, the MDBC is recommended.

## Introduction

Evidence-based practice is essential for any clinical-related work. However, when studies provide contradictory findings, it is difficult for clinicians to select appropriate techniques for clinical practice. To overcome this problem, systematic reviews and meta-analysis combining the results of multiple studies have gained popularity among researchers and the clinical community. 1 However, erroneous interpretations are possible when studies of differing qualities are combined and analyzed Hence, it has been recommended to assess the quality of studies selected for meta-research using standardized tools. 3 Several checklists/scales for quality analysis have been developed, with them being designed to analyze specific types of research studies. The checklists/scales developed to evaluate randomized control trials include the Cochrane risk of bias tool 2 4 , the Detsky Quality Assessment Scale 5 , the Consolidated Standards of Reporting Trials (CONSORT), 6 and the Jadad scale. 7 On the other hand, for analyzing the quality of observational studies, the scales developed were the Newcastle-Ottawa Scale 8 , and the Strengthening the Reporting of Observational Studies in Epidemiology (STROBE) 9 . The scales developed to evaluate any form of research were the Standard quality assessment criteria (SQAC) for evaluating primary research papers from a variety of fields, 10 and the Modified Downs and Black Checklist (MDBC). 11

Although several tools have been developed for the purpose of quality analysis of research articles, little information is available regarding which of the scales/checklists would provide more useful outputs, specifically in the field of audiology. Generally, the scales/checklists for quality analysis are randomly selected, if they meet the general purpose of the study. It is usually presumed that a scale/checklist that has more items would be more appropriate, without any actual knowledge of whether this is true. Hence, there is a need to compare the utility of scales/checklists that have a similar purpose, but with varying items.

The current study aimed to compare quality analysis carried out using the SQAC and MDBC for a set of articles in APD) in older adults. We also aimed to compare the inter-judge reliability of researchers with a dissimilar number of years of experience.

## Methods

A list of 102 keywords/phrases was initially made for the literature search on APD in older adults. These words/phrases were synonyms of three broad subareas (auditory processing, older adults, and hearing/hearing loss). The initial number of keywords/phrases was shortlisted to 77 by two professionals in the field having a minimum of 10 years of research/clinical experience, who were instructed to eliminate words/phrases that they felt were redundant or irrelevant. Out of the 77 shortlisted keywords/phrases, 42 were related to auditory processing, 12 to the term *older adults* , and 23 to the terms *hearing* / *hearing loss* . The customized search syntax was used for each search engine using these following key words: *temporal* , *duration'* *auditory processing* , *masking* , *period* , *span* , *time* , *term* , *continuation* , *patterning* , *gap detection* , *auditory* *discrimination* , *asynchrony* *detection* , *speech* *in* , *localization* , *lateralization* , *binaural* , *figure ground discrimination* , *auditory memory* , *auditory sequencing* , *auditory stream segregation* , *auditory integration* , *auditory separation* , *monoaural separation* , *low redundancy test* , *gap in noise* , *duration pattern* , *dichotic* , *time* *compressed speech* , *reverberant speech* , *masking level difference* , *auditory closure* , *degraded acoustic signal* , *screening central auditory processing* , *cognitive processing* , *higher order* , *checklist* , *geriatrics* , *older adults* , *elderly* , *pensioner* , *senior citizen* , *aged* , *senior* , *oldster* , *veteran* , *elders* , *old person* , *super annuitant* , *gerontology* , *hearing* , *hearing loss* , *presbyacusis* , *extreme* , *greater than 2* , *high* , *pitch* , *frequency* , *octave* , and *extended* . Using these keywords, articles on the topic of APD in older adults were identified through specific search engines and databases (PubMed/MEDLINE, Google Scholar, Cochrane, DARE, Psychinfo, and Ingenta Connect).

The articles were retained only if they studied at least one auditory process. The articles were shortlisted irrespective of age, health, and hearing status of the participants studied, to avoid unintentional exclusion. Those titles that did not specify the population were also selected. Thus, 88 articles were shortlisted for the next level of screening after removing duplicates. The abstracts of these 88 selected articles were scrutinized independently by two audiologists with experience in auditory processing. Both audiologists utilized the following guidelines to retain articles for further scrutiny:

The study was not a review of the literature;
The study was conducted on human participants;
The study had at least one group of participants with or without mentioning the age group;
The study addressed auditory processing in general or a specific auditory process;
The participants had normal hearing sensitivity or only high-frequency hearing loss;
The article was available in English.

**Fig. 1:**
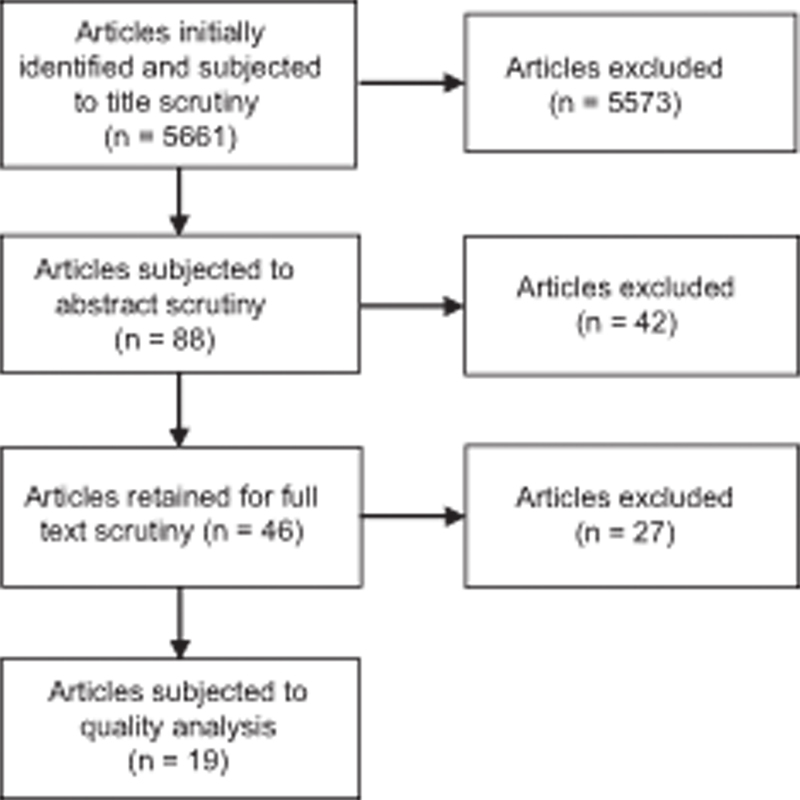
Flowchart of the literature review process and its outcomes.

The article abstracts that did not mention the sample size were also retained for further scrutiny. Of the 88 abstracts, 42 were excluded for not satisfying the above-mentioned inclusion criteria. The full texts of the remaining 46 articles were retrieved and reviewed by the audiologists. After reviewing the full-length articles, 27 of them were excluded as they did not study older adults, or any auditory process, as well as included participants with hearing loss in the low frequencies also. Thus, a total of 19 articles that met the inclusion criteria were selected for the quality analysis. Fig. 1 shows the process and outcomes of the literature review conducted.

### Materials

The two checklists, SQAC and MDBC, were used to carry out the quality analysis of 19 articles in APD in older adults. These checklists were selected as they were designed to be used for any type/quantitative research. The SQAC consisted of 14 questions with no subdivisions, while the MDBC consisted of 27 questions (10 about reporting, 3 about external validity, 7 about biases in the measurements, 6 about biases in the subject selection, and 1 about statistical power). The scoring pattern used for both checklists was as recommended by the authors. 10 11 Thus, each question of the SQAC was assigned a score of 2, while each question of the MDBC carried a score of 1 except for the 5 ^th^ question, which carried a score of 2, resulting in both checklists having a maximum score of 28.

**Fig. 2:**
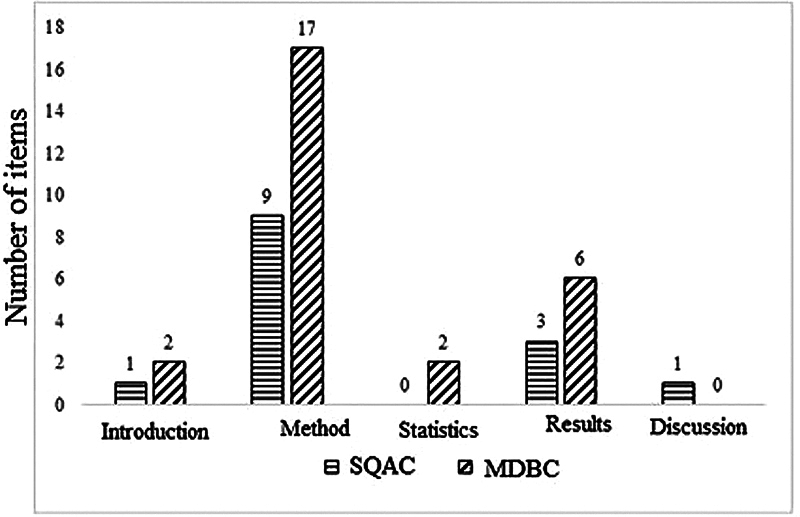
Number of items under different subsections of the Standard quality assessment criteria (SQAC) for evaluating primary research papers from a variety of fields and Modified Downs and Black Checklist (MDBC).

To equate the two checklists for the purpose of analyses, the items in both tools were classified by the investigators under five subsections (introduction, methods, statistics, reporting of results, & discussion) to demarcate the different sections in the research articles ( Fig. 2 ). The classification of the items under the 5 subsections was done by 4 independent researchers with a minimum of 12 years of experience in the field of audiology. The categorization of the items was accepted if the responses of at least three of the four researchers agreed. From the categorized items of the 2 checklists depicted in Fig. 2 , it can be observed that the SQAC had no items related to statistics, while the MDBC had no items related to discussion. Each of the other three subsections in both checklists (introduction, methods, and reporting of results) had similar maximum possible scores ( Table 1 ).

**Table 1:** Scores for the subsections of the Standard quality assessment criteria for evaluating primary research papers from a variety of fields and Modified Downs and Black Checklist

Subsections	Maximum possible score for the SQAC	Maximum possible score for the MDBC
Introduction	2	2
Method (participant selection, controlling the variables)	18	18 ^#^
Statistics	0	2
Reporting of results	6	6
Discussion	2	0
Total	28	28

**Abbreviations:** MDBC, Modified Downs and Black Checklist; SQAC, standard quality assessment criteria.

Note: ^#^ One question carried a score of two and the remaining a score of one.

### Procedure

The quality analysis of the selected articles was done using both the checklists selected (SQAC and MDBC). Each of the 19 selected articles was scored by 2 researchers, one having 5 years of experience and the other having 13 years of experience in the field of audiology. A double-blind rating was done, in which the two researchers were unaware of each other's ratings. Also, the order in which they chose the articles and tools was randomized so that there was no order effect. They were required to score each article for all five subsections (introduction, methods, statistics, reporting of results, and discussion).

### Statistical Analyses

Statistical analyses were performed using the IBM SPSS Statistics for Windows, version 23.0 (IBM Corp., Armonk, NY, USA). The Shapiro-Wilk test of normality indicated that the data were non-normally distributed ( *p* < 0.05), hence nonparametric statistics were done. Both descriptive and inferential statistics were carried out.

## Results

The data were analyzed to check for significance of difference between the two rating scores of the articles when evaluated using the two checklists. Additionally, the correlation between the scores obtained using the two checklists was also obtained. Prior to these analyses, the interrater reliability was assessed. It is evident from Table 2 that the scores assigned by the two experimenters for each of the subsections of the two tools were similar. A two-way random effects model inter-class correlation confirmed the presence of a good interrater reliability ( *r* = 0.89), based on the criteria given by Koo and Li. 12

**Table 2:** Scores assigned by each experimenter (exp) for the subsections of two tools used [Standard quality assessment criteria for evaluating primary research papers from a variety of fields & Modified Downs and Black Checklist

	Introduction		Method		Statistics		Reporting of results		Discussion		Total	
	Exp 1	Exp 2	Exp 1	Exp 2	Exp 1	Exp 2	Exp 1	Exp 2	Exp 1	Exp 2	Exp 1	Exp 2
SQAC	28	28	176	179	−	−	86	89	34	34	324	330
MDBC	38	38	200	203	19	20	54	56	−	−	311	317

**Abbreviations:** MDBC, Modified Downs and Black Checklist; SQAC, Standard quality assessment criteria.

As the interrater reliability was good, the scores of the two researchers were combined for each of the subsections ( Table 3 ). From Table 3 it can be observed that the same 19 articles had lower mean/median scores with greater variability for the introduction and method when rated using the SQAC compared to the MDBC. However, for reporting of results, it was lower for the MDBC compared to the SQAC.

**Table 3:** Mean, median, standard deviation, and range for the combined scores of the two researchers for the subsections and total scores of the Standard quality assessment criteria for evaluating primary research papers from a variety of fields and Modified Downs and Black Checklist

Subsections	Introduction		Method		Statistics		Reporting of results		Discussion		Total	
Checklists	SQAC	MDBC	SQAC	MDBC	SQAC	MDBC	SQAC	MDBC	SQAC	MDBC	SQAC	MDBC
Mean	2.94	4	18.68	21.21	0	2.05	9.21	5.78	3.57	0	34.4	33.04
SD	1.03	0	2.56	1.13	0	0.23	1.68	1.27	0.76	0	6.03	2.63
Median	2	4	19	21	0	2	10	6	4	0	35	33
Range	2	0	9	5	0	1	6	5	2	0	19	11
Maximum possible combined scores	4	4	36	36	0	4	12	12	4	0	56	56

**Abbreviations:** MDBC, Modified Downs and Black Checklist; SD, standard deviation; SQAC, Standard quality assessment criteria.

The significance of difference between the two checklists was assessed for the three subsections that had equivalent scores (introduction, methods, and reporting of results) and for the total score using Mann-Whitney U test. Compared to the SQAC, significantly higher scores were seen using the MDBC for the introduction and method subsections, but it was significantly lower for the reporting of results subsection and the total score.

Furthermore, no significant correlation between the scores of the two checklists was observed, when measured using Spearman's rank correlation. This was observed for each of the subsections that had equivalent scores (introduction, methods, and reporting of results) as well as the total scores ( Table 4 ).

**Table 4:** Significance of difference and correlation of the scores of the subsections having equivalent scores and the total scores of the two checklists (standard quality assessment criteria for evaluating primary research papers from a variety of fields & Modified Downs and Black Checklist)

Sections	Significance of difference		Correlation	
	*Z*	*p*	*r*	*p*
Introduction	-3.63	0.00	−	−
Method	-3.43	0.01	0.33	0.17
Reporting of results	-4.70	0.00	-0.10	0.68
Total	-2.50	0.01	0.17	0.31

## Discussion

The good interrater reliability of the current study for both checklists indicates that a researcher who has just 5 years of experience can utilize both tools as reliably as a more experienced researcher. This is probably an indication of the unambiguous nature of the items in both checklists enabling the researchers to rate the articles appropriately. Although the MDBC was more complex than is SQAC, the way the items within the checklist were framed perhaps enabled a relatively naïve researcher to use it with ease.

Moreover, from the descriptive statistics provided in Table 3 , it can be inferred that due to the higher variability seen in SQAC compared to MDBC, the former checklist would be able to distinguish the quality of articles more effectively. The variability indicates that both researchers rated the quality of some articles much lower while that of other articles much higher when using the SQAC. This higher variability was observed for all three subsections that were compared across the checklists (introduction, methods, and reporting of results) and the total score. Thus, the SQAC could differentiate the quality of articles not only based on the total scores, but also with reference to the introduction, methods, and results. On the other hand, using the MDBC, the researchers tended to judge the articles as being similar. The two researchers who judged the 19 articles were also of the opinion that the quality of the articles varied and could not be grouped as being similar. Thus, their opinion was better reflected by the scores of the SQAC than of the MDBC. This difference in the outcome of the checklists may be due to the focus of the items included in each of them. The items in the SQAC focused majorly on general research, which include studies related to diagnosis as well as intervention. On the other hand, the MDBC had several items regarding intervention (11 questions), unlike the SQAC (3 questions). All of the 19 studies that were evaluated using these tools focused on the evaluation of auditory processing skills. Hence, it is construed that if the quality of articles dealing with intervention are to be studied, the MDBC may also be able to differentiate them.

The lack of correlation between the scores of the two checklists also substantiates that they do not evaluate similar aspects. Additionally, it was noted that while the SQAC had one question regarding the discussion of the studies, which is an important component of any study, the MDBC did not have any. Although the SQAC did not have any questions on statistics, unlike MDBC, which had two questions, the former checklist indirectly tapped it in the reporting of results subsection.

Thus, from the above, it can be noted that both checklists have their specific utility. Depending on the focus of the research study on quality analysis, the appropriate checklist should be selected.

## Conclusion

Based on the findings of the study, the SQAC appeared to differentiate the quality of articles better than the MDBC. However, it was noted that the MDBC had items that could assist researchers to examine factors related to external validity, internal biases, subject selection, reporting, and statistical power. Hence, the selection of an appropriate quality assessment checklist depends on the objective of the analysis. If the aim of a quality analysis study is to differentiate articles based on their overall caliber, it is recommended that the SQAC be used. However, if the aim of a quality analysis study is to distinguish research articles primarily based on the control of variables, or differentiate intervention-based studies, the MDBC is recommended.
